# Resveratrol and Reproductive Health

**DOI:** 10.3390/life12020294

**Published:** 2022-02-16

**Authors:** Radmila Novakovic, Jovana Rajkovic, Milos Gostimirovic, Ljiljana Gojkovic-Bukarica, Nebojsa Radunovic

**Affiliations:** 1 Institute of Pharmacology, Medical Faculty, Clinical Pharmacology and Toxicology, University of Belgrade, 11129 Belgrade, Serbia; jovana.rajkovic@med.bg.ac.rs (J.R.); milos.gostimirovic@med.bg.ac.rs (M.G.); ljiljana.gojkovic-bukarica@med.bg.ac.rs (L.G.-B.); 2 National Laboratory for Molecular Diagnostics, Department of Microbiology, University Clinical Centre of Serbia, 11129 Belgrade, Serbia; 3 Clinical Hospital Center “Dr. Dragisa Misović-Dedinje”, 11129 Belgrade, Serbia; 4 Department of Medical Sciences, Serbian Academy of Sciences and Arts, 11129 Belgrade, Serbia; nebojsa.radunovic@med.bg.ac.rs

**Keywords:** resveratrol, polyphenols, dysmenorrhea, endometriosis, polycystic ovary syndrome, uterine contractility, pregnancy, umbilical blood vessel, embryogenesis, phytoestrogen

## Abstract

Resveratrol (RSV), a plant-derived polyphenol, demonstrates broad-spectrum health benefits, including anti-proliferative, anti-inflammatory, antidiabetic, anti-ischemic and antioxidant effects. The aim of this review is to give an important heads-up regarding the influence of RSV as a phytoestrogen, RSV effects on most common pregnancy-related complications, as well as its impact on the embryogenesis, spermatogenesis, and women’s reproductive health. Considering the important implications of RSV on human reproductive health, this overview could provide a groundwork, encouraging more detailed research at the clinical level.

## 1. Introduction

Polyphenols are one of the main classes of secondary phytometabolites, chemically characterized by the series of phenolic structural units. They are in the basis of specific physical, chemical and biological properties of the plants. Polyphenols have been studied extensively over the past decade as the adjuvant agents, in terms of attenuating risk factors for cardiovascular disease, diabetes, malignancies, neurodegenerative disorders, metabolic syndrome and obesity.

One of the most studied natural polyphenol, with health benefits clearly established in various in vitro and in vivo models, as well as in clinical studies, is resveratrol (RSV). RSV (3, 5,4′–trihydroxystilbene) belongs to a class of dietary stilbenes and it is contained in more than 70 types of plants and their products, especially in red wine [1]. Recent studies indicate that RSV shows wide spectra of well-established health properties, such as anti-inflammatory, antioxidant, anti-carcinogenic, anti-aging, neuroprotective and cardioprotective [2]. Preclinical studies of RSV pharmacodynamics have identified numerous molecules and cell structures as sites of its action; consequently, for a long time it has been considered as “one molecule—many targets”. It affects some of its targets directly, while others tend to be modulated indirectly, through changes in their expression levels. Over 20 molecules have been identified to which RSV binds directly [2]. On the other hand, RSV’s antioxidant capacity, bioavailability and pharmacokinetics (the extent and the dynamics of absorption, distribution within the body, metabolism and elimination), can influence its full bioactivity.

Sedentary lifestyle, fast food intake, and hereditary of genetic load are characteristics of modern life. In the last decades, pharmaceutical industries more often consider plant-derived molecules in order to develop alternative (or additional) therapy. Among plant-derived molecules, RSV has been identified as a promising agent in improving human health. Due to its mammalian estrogen-like chemical characteristics (structure, polarity, molecular weight), this substance has been categorized as phytoestrogen. Also, this “estrogenic activity” is related to the impact on enzymes that participate in the metabolism of endogenous hormones.

According to the promising results from animal studies and clinical trials, RSV has been considered as an effective agent in the treatment of the most common diseases related with women reproductive function, like endometriosis, dysmenorrhea, or polycystic ovary syndrome. Further, it was observed that RSV may have effects on different phases of pregnancy and fetal life, although there is evidence that its impact on the implantation is dependent on the menstrual cycle in which resveratrol supplementation occurs. Also, it may promote ovarian function without adversely impacting in embryo implantation during the proliferative phase of a menstrual cycle. In contrast, after the initial phase, it may promote decidualization due to its antisenescence activity. In order to mitigate its adverse reproductive outcomes, it is important to align resveratrol supplementation to the phase of a menstrual cycle.

Preventive effects of RSV on pregnancy-related complications have been observed in pre-clinical and clinical studies. The different study designs, as well as the large variability in the dose, route and courses of RSV administration, make difficult to reach strong conclusions about the health benefits of RSV as a phytoestrogen. If we take into consideration a common opinion, that is, if a health product is made from natural substances, then it is safe for human consumption, the question is: is this applicable in every condition? For a public use, different polyphenol (including RSV)-rich foods are being developed in various shapes: as nutraceutical food, new dietary supplements, natural health products, complementary, phytomedicine as well as alternative medicine products. This commercial availability has been increasing the interest of the general population in RSV intake through various sources [3]. However, since RSV is categorized as phytoestrogen, this can lead to more interesting research topics on its effects on a woman’s reproductive health, especially in pregnancy or postmenopausal life.

Taking all this into account, the most sensitive target group for the use of dietary polyphenols and its effects are probably pregnant women, considering the importance of proper fetal growth and development. Therefore, it is extremely important to understand the influence of maternal polyphenol consumption (especially RSV), its possible benefits and/or negative effects on a human reproductive health, pregnancy and fetal health.

This article aims to review RSV pharmacology and to summarize its possible beneficial and/or adverse effects on reproductive health, focusing on understanding the relevance of RSV in the adult reproductive system and in fetal tissues. Considering the importance of the reproductive physiology and medicine overall, the review also aspires to underline the role of RSV in a few different conditions and levels: hormonal action as a phytoestrogen, most common pregnancy-related complications (pre-eclampsia and gestational diabetes), RSV effects on human umbilical blood vessels, RSV influence on the embryogenesis and spermatogenesis and its effects on women’s reproductive health.

## 2. Methods

A literature search was performed using the National Center for Biotechnology Information (NCBI) PubMed database and Scopus database. The period from 2000–2021 was covered in this search and is focused only on papers published in English. Relevant keywords (polyphenols, resveratrol, diet, phytoestrogens, pregnancy, preeclampsia, gestational diabetes, umbilical blood vessels, embryogenesis, spermatogenesis, endometrium, dysmenorrhea, endometriosis, PCOS) were used to gather information regarding influence and effects of RSV on pregnancy-related complication, embryogenesis and spermatogenesis and women reproductive health.

## 3. RSV as a Phytoestrogen

Phytoestrogens are naturally-derived plant compounds, which name indicates the similarity with hormone estrogen. All phytoestrogens are divided into three groups: polyphenols, flavonoids and isoflavonoides. Phytoestrogens, due to structural and functional homology with estrogen (17 beta-estradiol benzoate), have been indicated as very important substances for human reproductive health. More than twenty years ago it was reported that RSV binds to and activates transcription by the estrogen receptor (ER) [4]. However, phytoestrogens are very weak ER agonists and are able to induce an agonistic effect only in very high doses [5]. Further research of RSV ER-dependent mechanism has pointed out that this mechanism includes ER α [6]. The most structural similarity is shown by RSV with diethylstilbesterol (DES), which has also greater affinity to the ER α [7]. Research performed on locus coeruleus (LC), a noradrenergic brain stem nucleus that is greater in females, showed that the application of RSV on rats of both sexes is leading to the decrease in the differences between sexes [8].

RSV has been taken in consideration as an important phytoestrogen due to its capacity, and is observed in vivo in different female mammalians to modulate ovarian function and steroidogenesis [9]. The exposure of pregnant mice to RSV (four daily s.c. injections of 0.5 or 10 mg/kg/day of RSV) significantly increased the percentage of time spent in the diestrus phase compared to untreated controls [10]. However, those observed effects on the reproductive tract, after high dose of RSV, were transient. Another study on rats performed by the same research group showed that prepubertal exposure to 100 mg/kg of RSV causes earlier vaginal opening [11]. Kubo et al. [8] investigated the effects of RSV exposure (1500 µg/kg/day) on rats of both sexes, showing that exposure of RSV on male rats did not affect their sexual development or sexual behavior, while in females, RSV caused delay of the day of vaginal opening. This observed property of RSV was in contrast with a well-known effect of the perinatal exposure to estrogen on the acceleration of the vaginal opening. This finding was interpreted as mixed RSV property to act as an ER agonist/antagonist, instead of only as an ER agonist [12]. In the same study [8], it was also observed that RSV-exposed female rats had a reduced number of normal cycles which correlates with observed abnormal estrus cycles. It was confirmed that the exposure to RSV during the fetal and suckling periods in female rats mainly affected the reproductive function.

In research on the gonadal intact and ovariectomized adult female rats, performed by Henry and Witt [13], it was confirmed that RSV acts as possible mixed agonist/antagonist. While in vivo application of RSV on intact adult female rats caused reduction in body weight, disruption of estrous phase, and ovarian hypertrophy, application of RSV (s.c. injection of 100–1000 µg) in ovariectomized rats did not show these effects. This can be explained by the fact that RSV acts as a weak estrogen (and, thus, partial ER agonist), blocks the ER when given in high doses and affects ovarian function, leading to a reduction in circulating estradiol levels, with a weight gain as a consequence (post-menopausal women are an example of this phenomenon). On the other hand, observed characteristic of RSV can be due to its dual effects as agonist and antagonist, which differs it from other phytoestrogens.

Sato et al. [14] also confirmed that 100 mg/kg RSV causes slightly earlier vaginal opening and prolongation of estrus phase. In the same study it was observed that RSV did not increase body weight. Also, they have shown that short RSV treatment in prepubertal female rats had effects on endocrine function and accelerates development of n-methyl-n-nitrosourea (MNU)-induced mammary carcinoma.

Besides proposing that RSV acts through an ER-dependent mechanism, its ER-independent mechanisms were observed, too. ER-independent mechanisms of RSV include different intracellular signaling mechanisms mediated by tyrosine kinases cascade (AMPK pathway), SIRT1 (by increasing expression of SIRT1) and eNOS [15,16]. Those effects have been reported in nanomolar concentrations.

Due to the confirmed impact on the female reproductive system and reproductive behavior, as well as on the different parts of the brain, there are a lot of controversies regarding to the consumption of the foods that contain phytoestrogens. However, a well-known French paradox popularizes the consumption of red wine due to its most important constituent—RSV. Second debatable observation regarding RSV is its low bioavailability in humans. However, studies undertaken on human umbilical vein endothelial cells (HUVEC) and in vivo on animals (rats) have shown that passive diffusion and active transport of RSV into vascular endothelium provides permanent availability of RSV to blood vessels, making the intracellular RSV more important compared to that available in serum [17].

## 4. RSV—Additional Roles on Overall Reproduction

Since RSV supplementation poses many systemic beneficial effects, it is justified to consider it as an important molecule with the promissive, positive contribution for successful embryogenesis and fetal development, as well. It is known that the impairment of female reproductive function can be caused by numerous spectra of diseases, the pathology of which lies in different metabolic and age-dependent changes of the organism. Nowadays, in the time of massive industrialization and constant physical stress, those diseases can strongly aggravate the ability of women to conceive uninterruptedly. Maintaining the natality rate within the acceptable ranges represents a reflection of the country’s economic stability; yet, at the same time, it shows weak points in the country’s health network. Also, pathology of advance-aged women, reproductive and postovulatory aging, together with social trends in delaying childbirth represent stumbling stones in preserving maternal and perinatal health.

### 4.1. The Effects of RSV on the Endometrium

**Figure 1:**
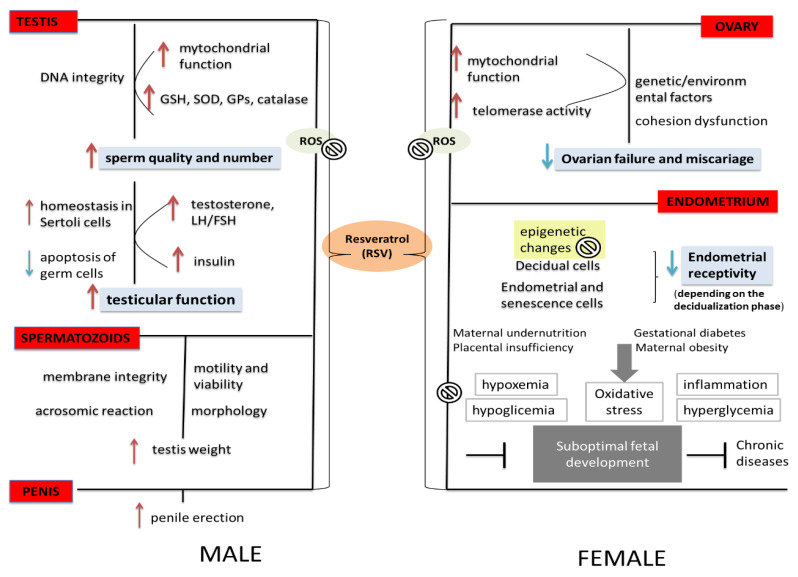
The diagram shows the effects of RSV on male and female human reproductive function. ROS—reactive oxygen species, GSH—glutathione, SOD—superoxide dismutase, RSV—resveratrol. Modified according to [29].

Beneficial effects of RSV on ovaries include activation of SIRT1 molecules. This activation leads to anti-oxidative effects, which protect oocytes from senescence-dependent damage [18]. Also, RSV-induced SIRT1 upregulation increases levels of LH (but not FSH) receptors and steroidogenic enzymes (aromatase) in the model of rat ovaries [19]. It also exerts its effects through GnRH-gonadotropin-ovarian gonadotropin receptor axis. These activities show a significant difference in aged women, since the decreased number and function of mitochondria are influencing total oxidative status of the cells. The decline in the ovarian function, thus, can be reversed by stimulation in mitochondrial number and function [20]. Except for anti-oxidative, other SIRT1-mediated RSV effects also pose an impact on oocyte maturation, and it is known that, besides oocytes, RSV has also the effect in the modulating genes of granulosa cells (Figure 1, right). Another study that applied an animal model of ovarian aging to examine the involvement of nuclear SIRT1 and SIRT6 as well as mitochondrial SIRT3 molecules on ovarian reserve and developmental potential has shown strong correlation between depleted expression of the SIRTs and ovarian dysfunction [19].

### 4.2. The Effects of RSV on the Embryogenesis—Preclinical Studies

The upregulation of mitochondrial biogenesis through SIRT1 molecule can also be important factor for preserving physiological process of embryogenesis as well [21], although some studies suggest these effects are dose-dependent and occur at higher RSV concentrations. In a study with aged mice, examining factors influencing embryonic development, the effects of RSV were compared to different media containing granulocyte-macrophage colony-stimulating factor (GM-CSF) and dichloroacetic acid (DCA), resulting in significantly greater developmental competence and pregnancy potential after blastocyst transfer (76%, 56%, 60.7% in RSV, GM-CSF and DCA, respectively; *p* < 0.05) in the RSV group. This is an important result, considering that GM-CSF is proven to improve pregnancy rates in patients with multiple failure in in vitro fertilization (IVF) and DCA could improve blastocyst development and mitochondrial activity in embryos produced from aged mice [22]. Compared to melatonin, RSV has shown a significantly lower fertilization rate, poorer quality of good-blastocyst formation and lack of difference in the pregnancy or miscarriage rate, compared to a control group [23]. In fact, during embryo transfer, RSV may act adversely on the decidualization process in spite of good competency of the transferred embryos [24]. Additionally, RSV activates calcium dependent SIRT1-modulated attachment of an embryo to the endometrium, and, due to its antiproliferative and antiapoptotic effects, prevents formation of an ectopic endometrium [18]. In a sheep animal model, nuclear maturation rate or intracellular oxidative status in cloned embryos have not been significantly improved by 0.5 µM concentrations of RSV [25]. On the contrary, oocyte quality and morulae compaction were positively affected, mostly due to RSV-modulated increase in the expression of calcium-dependent cell adhesion molecules, required for morulae compaction [22,25]. Moreover, RSV may reverse hyperleptinemia, improve hypothalamic leptin signaling and cause epigenetic changes by hypermethylation and consequent reduction in BRCA1 expression in the tissue of rat offspring [26]. Besides, animal studies that examined the relationship between maternal polyphenol consumption during the late pregnancy and antenatal circulation have resulted in constriction of ductus arteriosus and consecutive dilated and hypertrophic right ventricles, probably due to prostaglandin inhibition [27].

Conversely, since the requirements for the successful pregnancy consist of decidualization (hence, trophoblast invasion) and activation of the local inflammatory response, due to other properties, RSV may suppress embryo implantation [18]. In fact, hormonal changes and consequent morphological events of the endometrium (decidualization of HESC), trophoblast invasion, cell differentiation and apoptosis) denoting the development of normal pregnancy [20] can be highly detrimented by RSV supplementation, and lead to an implantation failure and pregnancy loss, mostly due to its antiapoptotic and antisenescence activity [18]. These properties of RSV represent the main disadvantage for the treatment of infertility, since normal decidualization entails apoptosis/senescence activity and consecutive differentiation of the cells. Additionally, retinoic acid (RA) activates signal pathways involving several regulatory proteins: cellular retinoic acid-binding protein 2 (CRABP2) and fatty acids binding protein 5 (FABP5,) which promote genes to cause cell-cycle arrest and peroxisome-proliferating activated receptor (PPAR), which promotes cell differentiation [28]. Normally, decidualization first increases and then decreases RA-PPAR signaling, while decidual cell secrete proapoptotic factors (p16 and p53) [18,28]. RSV leads to downregulation of CRABP2-RAR signaling, which suppresses the expression of p53 and causes implantation failure, along with the inhibition of prolactin (PRL) induction (a widely used decidual marker gene) and IGFBP1 (insulin-like growth factor-binding protein-1) [29]. Moreover, epigenetic changes (acetylation of regions in decidual markers) represent important association with decidualization. Through SIRT1, RSV induces deacetylation of decidua-related genes, thus decreasing receptivity of the endometrium [19]. According to Mestre Citrinovitz et al. [18], RSV enhances the decidualization of the human endometrial stromal cells (HESC) following protocol using 6.25–50 µM ranges, after treating HESC with cyclic AMP (cAMP), estradiol (E2) and medroxyprogesterone acetate (MPA) from the third day of decidualization. In a different study [30], after exposing HESCs to RSV and treating with progesterone and cAMP from the start of decidualization, it was concluded that RSV inhibits decidual transformation. However, those studies were using protocols that are different in the timing of RSV treatment during the process of decidualization, and major conclusions can be attributable whether RSV supplementation occurred during the initial proinflammatory phase or after the initial phase of decidualization.

### 4.3. The Effects of RSV on the Embryogenesis—Clinical Studies

In the area of clinical study, a similar outcome as in the preclinical studies (live-born infants, the number and competence of mature oocytes and embryos) was investigated in the population of women receiving an IVF treatment [31]. This study investigated the association between RSV and IVF outcome, compared to independent action of SIRT1, SIRT3 and SIRT6. Unexpectedly, it was revealed that RSV seemingly did not have the role in oocyte maturation and clinical pregnancy in IVF [18,31]. The reason for such result was probably insufficient concentrations of RSV to cause dose-dependent stimulation of SIRT1 expression [31]. Additionally, RSV exerts beneficial effects in the courses of complicated pregnancies (gestational diabetes, maternal undernutrition/obesity, teratogen exposure, placental insufficiency), improving suboptimal fetal development (macrosomia/intrauterine growth retardation), and the efficacy of the oxygen and nutrients transfer through placenta, making an impact on lifelong health [32]. It was also shown that a polyphenol-rich diet could alter fetal ductal dynamic, and the recommendation for maternal polyphenol intake was made correspondingly. Studies confirming this statement are numerous, stating that polyphenol cessation for two or more weeks in the third semester improves ductal flow dynamic and decrease right ventricle in normal fetuses [32,33]. Currently, there are many studies that show association of different circulatory compartments and polyphenol intake, so further steps were made in investigating the role of RSV in the neonatal circulation [30]. Notwithstanding, dietary polyphenol restriction is surely accompanied by increase in plasma levels of PGE_2_ and reversion of fetal ductal constriction [33].

Additionally, results from a recent clinical study aimed to compare clinical pregnancy rate and the risk of miscarriage between women using RSV (200 mg daily continuously) and women in a control group, have suggested a decrease in the clinical pregnancy and increased risk of miscarriage in RSV group [34]. Evaluating the effect of RSV on pregnancy outcome in IVF-embryo transfer in infertile women, this study concluded that if tissue concentration of RSV decreased before decidualization, negative RSV effects on the implantation would not be so prominent. In fact, low adverse effects on decidualization and implantation are associated with the cessation of RSV intake at the beginning of the luteal phase [31].

RCT using RSV for 3 months have shown not only improved fertilization rate, but also improved number of oocytes and MII (metaphase II) oocytes, cleavage embryos, blastocytes and cryopreserved embryos. No significant differences in biochemical or clinical pregnancy, live birth, and miscarriage rates were revealed in the protocol without RSV treatment during luteal phase [35]. Since decidualization is a multistep process, depending on the protocols used, it seems that apparent effects of RSV on endometrium is dependent on its phase—antagonizing decidualization by inhibiting the obligatory proinflammatory phase or promoting it by restraining decidual senescence [36].

## 5. The Effects of RSV on Male Fertility

It seems that the antioxidative capacity of RSV plays a most important role in the preservation of male fertility. The acceptable level of the oxidative stress within the cell is maintained through the activation of AMPK signal pathway and consequent improvement of the basal oxidative status. Since the extensive amounts of ROS are formed during the cellular respiration withing mitochondria, it is suggestive that many polyphenolic compounds (such as RSV) will exert their antioxidative capacity mainly through modification of the mitochondria function. In fact, recently published articles have confirmed strong correlation between the parameters of mitochondria function (membrane integrity, respiratory rate activity, ROS production) and the human sperm quality [37], which make mitochondria key organelles for the improvement in semen viability and maturation. Much of this beneficial action is mediated through AMP-dependent protein kinase signal pathway, depicting the most important signaling activated by RSV. Either through reduction of the ROS production in the mitochondria, inhibition of the lipid peroxidation or through increasing the expression of the essential antioxidative enzymes, glutathione peroxidase (GPs), catalase and SOD, RSV may act on different steps in the antioxidative protection. All these factors together, with the addition of direct protective effect of RSV on spermatogenesis, are important in preserving DNA integrity and the viability of the male reproductive cells [38]. On the other hand, some studies revealed pro-oxidant effects on mitochondria after high-dose RSV supplementation, concluding that RSV in different concentrations can differently affect mitochondrial function [39]. Nevertheless, RSV significantly improves semen quality when added to a cryopreservation medium [40]. Although cryopreservation of human semen is important in reproductive medicine, it can damage sperm, mostly due to ROS overproduction during the lipid peroxidation. In the study, RSV was able to prevent oxidative damage, but the loss of sperm motility has not been prevented [40]. This is probably attributed to the anti-inflammatory properties of RSV, considering the role of prostaglandins in the sperm motility. This statement is confirmed in many experimental models, although the exact mechanism is yet to be established. In the clinical studies, effects of 30 µM of RSV supplementation on fertility in obese males with astenospermia have shown improvement and protection in semen quality (motility, viability, acrosomal reaction). Research has concluded that RSV affects male fertility, not only through hormonal changes but also by directly improving sperm function (Figure 1, left) [38].

## 6. The Effects of RSV on Complications during Pregnancy

The effects of RSV on the cardiovascular system (CVS) and blood vessels are very well documented and popularized, but how much do we know about RSV’s effects on umbilical blood vessels? It has been confirmed that RSV can relax human and animals blood vessels. However, umbilical blood vessels are different than the other types of the blood vessels in the organisms. Perhaps, the most important difference is that umbilical vein transports oxygenated, nutrient-rich blood, while the umbilical artery carries deoxygenated blood. Human umbilical cord contains two umbilical arteries and one umbilical vein, but in small percentage (1–5%), a cord with one artery and one vein has been detected too.

Interestingly, most of the confirmed RSV benefits on CVS derive from in vitro research of RSV effects on HUVEC. The experiments on this cell culture indicated RSV’s anti-inflammatory, anticoagulation, and antifibrinolytic effects [41]. The application of different concentrations of RSV on cultured HUVEC decreases the concentrations of von Willebrand factor (VWF), tissue plasminogen activator-1 (t-PA-1), secretion of IL-8 and diminishes the activity of factor VIII, which are all representative markers of coagulation, fibrinolysis and inflammation. It has also been shown that RSV reduces mRNA expression levels of VWF and t-PA-1. The application of RSV on HUVEC also demonstrates the possible role of RSV in the prevention of venous thrombosis [42]. It is observed that RSV depresses the expression of thrombosis-associated markers induced by hydrogen peroxide (H_2_O_2_).

Also, RSV on HUVEC significantly inhibits all oxidative alternations caused by mitochondria-derived oxidative damage, and subsequently protects HUVECs from apoptosis [43]. In a study undertaken by Yang at el. on HUVEC, it was proposed that RSV attenuates endothelial oxidative injury by regulating mitochondrial fusion via TyrRS-PARP1 signal pathway [44]. Pan et al. also performed a study on HUVEC and they proposed that the anti-inflammatory and anti-oxidative stress activities of RSV are mediated by repression of the p38 MAPK/NF-κB pathway and ROS production in the TNF-α-treated HUVECs [15].

Previously reported effects of RSV on HUVEC, but as well on the other cell lines and tissues are very important for some conditions that are correlated with women and pregnancy. One of the most frequently serious complications during pregnancy is preeclampsia. The exact cause of preeclampsia is still unclear, but it is proposed that abnormally implanted placenta leads to hypoxia and, consequently, increases oxidative stress and the release of the anti-angiogenic proteins along with inflammatory mediators. All of these cause endothelial dysfunction in the final step, and high blood pressure is the first symptom in pregnant woman. Treatment of HUVEC with RSV decreases the anti-angiogenic molecules of pre-eclampsia (such as soluble FMS-like tyrosine kinase 1, sFlt-1) and soluble endoglin (eEng) and in a dose dependent manner reduces ET-1 secretion and phosphorylation of eNOS [45]. Currently approved, and first-line, drugs in the treatment of preeclampsia are labetalol, nifedipine and hydralazine. Recently performed meta-analysis has indicated that the investigated combination of nifedipine/RSV requires a significantly shorter time to achieve the target blood pressure than hydralazine as a monotherapy. However, as nifedipine requires fewer doses than hydralazine to achieve the target blood pressure, the observed advantages with such drug combinations do not have great clinical importance [46].

Furthermore, RSV has been suggested as an agent for the treatment of diabetic complications in pregnancy. It is well known that RSV has anti-diabetic effects, and these effects have been confirmed in pregnant rodents as well. In pregnant diabetic dams where diabetes was induced with streptozotocin, RSV was able to decrease blood glucose level and to significantly decrease the cholesterol and triglycerides concentrations in the serum [46]. Also, it is known that diabetes may cause the disruption in the embryonal development and organogenesis, as it is the most sensitive phase of the development. Potential explanation for the observed embryonal malformation in diabetic dams is hyperglycemia-induced oxidative stress that leads to apoptosis. Consumption of RSV (100 mg/kg body weight) by diabetic dams may attenuate diabetes-induced oxidative stress, which consequently reduces the activated caspases and apoptosis, and the embryonic malformations could be avoided [47]. Due to these effects, Singh et al. [48] suggested further preclinical studies with RSV or RSV in combination with insulin and folic acid for the use during diabetic pregnancies and for nursing mothers.

Despite the fact that the uterine artery is not part of the umbilical blood vessels, the research on this blood vessel is highly important regarding pregnancy and fetal development. The study that was undertaken on pregnant sheep indicates positive correlation between absolute uterine artery blood flow, umbilical vein oxygen saturation, absolute fetal oxygen delivery and fetal growth [49]. In this study, twin-bearing Merino ewes (*n* = 6) were treated with one s.c. injection of RSV and vehicle and changes were observed by magnetic resonance imaging. In previous research undertaken by the same group of authors on the same animal model, using the same methodology, sustained RSV concentrations was confirmed in maternal plasma, while RSV was not detected in fetal circulation [50]. The possible effect of RSV on a fetal weight gain without its delivery in fetal circulation is promising data in different pregnancy conditions in which fetal growth and oxygen delivery are compromised.

The well-known benefit of RSV on relaxation of the blood vessels was confirmed on the human umbilical vein also. Proposed mechanisms of dilatation of the human umbilical vein without endothelium include acting on different potassium channels, but the authors stated that potassium-channels’ independent effects could not be excluded [51,52].

## 7. The Effects of RSV on Women’s Reproductive Health

A large percentage of women (>70%) suffer from uterine disorders, such as adenomyosis, premenstrual pain, dysmenorrhea and endometriosis. The endometriosis and adenomyosis affect different parts of the body, share some symptoms, and may require different treatments. In endometriosis, the same type of cells that line the uterus also grows outside of it, and that growth can breach nearby organs, such as fallopian tubes and bladder, causing complications. Adenomyosis, on the other hand, happens when the same kind of cells that line the uterus also grow deep in the muscular wall of the uterus and thickens it. The etiology of these disorders remains elusive, despite high prevalence. The clinical presentation of endometriosis is variable and includes some severe symptoms: dyspareunia, chronic pelvic pain, dysmenorrhea and subfertility or infertility [53]. There are proofs that this disorder is an inflammatory process that happens inside the pelvic cavity. For example, it is known that chronic inflammation has a significant role in the development and progression of the disease and various studies report increased concentrations of proinflammatory factors in the peritoneal fluid of the patients with endometriosis [54].

Based of the anti-proliferative, anti-inflammatory, anti-neoplastic and antioxidant properties, RSV is proposed as the potential agent to treat endometriosis. Data from in vitro studies suggest that RSV reduces invasiveness of endometriotic stromal cells, suppresses their inflammatory responses [55], increases the apoptotic index in epithelial cells of endometriotic-like lesions, induces reduction in human eutopic endometrium cell proliferation and increases apoptosis in primary cells cultures [56]. Furthermore, in the animal models of endometriosis, RSV supplementation display beneficial results, as it can decrease the number and volume of endometrial implants, suppress proliferation, vascularization, inflammation. [57]. However, it should be noted that the doses of RSV used in experiments on animals were quite high (10 mg/kg and 100 mg/kg) [57]. According to the data from human clinical studies, RSV can suppress growth and proliferation of endometriotic implants by reducing the production and activation of growth factors [55] and inducing the apoptosis (increasing the proapoptotic factors and decreasing the antiapoptotic) [58]. It has been shown that RSV can inhibit the invasion, adhesion, and angiogenesis of endometriotic ectopic lesions [59] and reduce the inflammation and oxidative stress, both in animal and human model study [58,60]. Arablou et al. study, conducted on 40 patients with endometriosis, have shown that the treatment of eutopic endometrial stromal cells and ectopic endometrial stromal cells with RSV can reduce the gene expression and production of insulin-like growth factor-1 (IGF-1), hepatocyte growth factor (HGF) and the gene expression of control endometrial stromal cells [61]. According to the previous studies, these two factors play important roles in the growth, proliferation, invasion, and angiogenesis of endometriotic implants. Furthermore, IGF-1 and HGF levels are higher in peritoneal fluid of women with endometriosis compared with the healthy women [62,63]. The effect of RSV was more remarkable in the ectopic endometrial stromal cells compared with eutopic endometrial stromal cells and control endometrial stromal cells. These findings suggest that RSV in a concentration of 100 µM could reduce the expression of IGF-1 and HGF in endometrial stromal cells, especially in ectopic endometrial stromal cells, which play a pivotal role in the disease progression [61]. Additionally, the effect of RSV on the management of endometriosis-related pain was investigated through clinical study in 12 patients who failed to obtain pain relief during the use of an oral contraceptive containing drospirenone and ethinylestradiol [62]. The addition of 30 mg of RSV to the contraceptive regimen resulted in a significant reduction in pain scores, with 82% of patients reporting complete resolution of dysmenorrhea and pelvic pain after 2 months of use. In a separate experiment, authors have shown that the inhibition of aromatase and cyclo-oxygenase-2 expression was significantly greater in the eutopic endometrium of patients using combined drospirenone and RSV therapy compared with the endometrium of patients using oral contraceptives alone. These results suggest that RSV potentiates the effect of oral contraceptives in the management of endometriosis-associated dysmenorrhea by further decreasing aromatase and cyclo-oxygenase-2 expression in the endometrium. This is of great significance since aromatase expression in the eutopic endometrial cells plays a crucial role in the clinical course of endometriosis [62].

**Figure 2:**
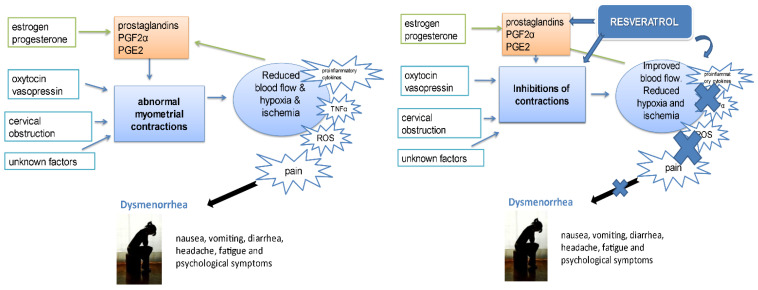
Scheme of some causes and consequences of dysmenorrhea. Increased levels of pro-inflammatory cytokines, interleukins and tumor necrosis factor-α (TNF-α), neutrophil hyperfunction and excessive reactive oxygen species (ROS) production have been reported in patients with dysmenorrhea. In the peripheral blood of patients with dysmenorrhea, it has been noted an increase in neutrophil function-dependent inflammatory metabolites, such as interleukins and prostaglandins. Due to decreased blood flow to the myometrium during uterine contraction, ischemia could happen. This can trigger the accumulation of ROS. Free radicals are the products of biological reduction reactions, and the overproduction of ROS has been implicated in the pathogenesis of dysmenorrhea. Proteins, lipids, DNA, and other molecules can be damaged and, in that way, ROS can cause disease and cell damage. ROS-induced changes to proteins and DNA can lead to altered cellular function or activation of proteolytic cascades that ultimately result in endometrial damage and inflammation. Antioxidants such as RSV protect cells from ROS damage and pro-inflammatory factors.

Dysmenorrhea is characterized by abdominal or lower back pain that lasts for at least two days during the menstrual cycle. It is very common in young women, with a prevalence of 45–95%, which varies among women from different countries [63]. It can be a consequence of endometriosis, but other causes, which etiology is not fully understood, may be questionable. These disorders may cause severe pain due to uncontrolled amplitude and frequency of the uterine contractions, particularly during the menstrual cycle. In addition to pain, uterine contractions may reduce uterine vascular flow and lead to hypoxia and ischemia. Dysmenorrhea is categorized as primary, representing the occurrence of menstrual pain in the absence of any apparent organic disorder, and secondary, which occurs in association with identifiable, functional, or structural organ impairment. Although primary dysmenorrhea is very common, its causes are partly unclear. Increased concentration of pro-inflammatory cytokines, interleukins, and tumor necrosis factor-α (TNF-α), neutrophil hyperfunction, and excessive reactive oxygen species (ROS) production have been reported in patients with primary dysmenorrhea [64]. Furthermore, severity of dysmenorrhea is directly related to the increased levels of prostaglandin F2α (PGF-2α), which repercusses the decreased uterine blood flow caused by the hypercontractility of myometrium and local contractions of the uterine vessels (Figure 2). Main pharmacological treatment of dysmenorrhea represents nonsteroidal anti-inflammatory drugs (NSAIDs), which, by inhibition of cyclooxygenase, suppress prostaglandins production. However, NSAIDs are not successful in all patients, and they have remarkable adverse effects, particularly on cardiovascular, renal and gastrointestinal systems. Oral contraceptives are usually administered to women who either do not respond or are intolerant to NSAIDs, but their use for treating dysmenorrhea has been a controversy [63].

The results from in vitro and in vivo animal model studies [65] indicate that RSV in concentrations 25–100 µM may be effective in inhibiting PGF-2α—induced uterine contraction. The doses from 0.5–2 mg/kg of RSV provoked relaxation of uterus in vivo. The authors of this study hypothesized that two glasses of RSV-rich red wine a day would reach an effective level in the blood for the treatment of hypercontractility induced by PGF-2α and additional consequences [65]. Correspondingly, results from the study on rat uterus have shown that RSV potently, in a concentration-dependent manner inhibits different models of contractions: spontaneous, induced with a low and high concentration of oxytocin [66]. Relaxation of uterine smooth muscle improves blood flow, reduces hypoxia and ischemia-induced accumulation of free radicals and proinflammatory factors and leads to decreased symptoms of dysmenorrhea [65,66]. Additionally, a study by Carreiro et al. [67] has shown regenerative and protective effects of RSV on non-pregnant murine uteri under hypoxia and described that these effects are mediated by blockade of ATP-sensitive potassium channels. Hypoxia causes a sharp decrease in the strength of many smooth muscles, including those in the uterus. Thus, RSV can act on both points—causing the relaxation of smooth muscles of uterus and blood vessels (which will improve blood flow) and reduce ischemia, decreasing the consequences of ROS. Furthermore, the retrospective analysis and prospective study of Ferrero et al. [68] examined the efficacy of oral administration of RSV on pain intensity and quality of life in women with primary dysmenorrhea. The study showed that RSV significantly decreases the intensity of dysmenorrhea symptoms and the need for analgesics pills in these patients. It has also significantly improved the quality of life of the study participants after 6 months of treatment [68]. In contrast, data from randomized, double-blinded, placebo-controlled clinical trial which included 44 subjects conducted to compare the effects of RSV (40 mg) with monophasic contraceptive pill have reported that the contraceptive pill was more potent than RSV in pain relief; the results also have concluded that RSV is not superior to placebo for the pain treatment in endometriosis [69]. Possible explanations for these differences in reported outcomes could be related to methodology, including the length of the treatment, pain score scales, doses and statistical analysis. It seems that RSV is a powerful agent, which can prevent or slow the progression of endometriosis, dysmenorrhea and their symptoms.

Polycystic ovary syndrome (PCOS) is characterized by hyperandrogenism, menstrual disorders and polycystic ovarian morphology. Patients with PCOS are at increased risk of type 2 diabetes, cardiovascular disease and infertility [70]. The mechanism of PCOS is not yet fully understood, but it is considered that insulin resistance and genetic factors play distinct roles in its pathophysiology. One of the proposed pathophysiological mechanisms recognizes PCOS as a susceptible genetic disorder, probably due to gene polymorphisms that potentially lead to the development of PCOS. Environmental factors, such as prenatal exposure to androgens, may also play role in the ontogeny of PCOS [71]. It is also associated with obesity, hypertension, ischemic heart disease, myocardial infarction, metabolic syndrome and non-alcoholic fatty liver disease [72]. Patients with PCOS more commonly display an atherogenic lipid profile: elevated total cholesterol, LDL and triglycerides, and lower HDL concentrations, which are risk factors for the aforementioned cardiovascular threats [73]. The multifactorial etiology of PCOS makes its treatment more demanding, and there is a constant need for new causative and effective modes of PCOS therapy. Treatment of those patients with RSV may represent a new and promising concept. Regarding this, most of the animal studies have reported beneficial effects of RSV on histomorphological features of ovaries, sex hormones and gonadotropins, glycemic control, inflammation and oxidative stress in PCOS patients. Also, in those patients, RSV ameliorated ovarian volume, quality of the oocytes (improving early embryonic survival), androgens and gonadotropins concentrations, levels of angiogenic factors and endoplasmic reticulum stress [74]. Banaszewska et al. evaluated RSV (1500 mg, *p.o.*) effects on 34 young PCOS patients in a double-blind, placebo-controlled trial over a period of 3 months. They observed a significant decrease in total testosterone (by 23%) and DHEAS (by 22%) as well as in fasting insulin levels (by 32%) in comparison to placebo [70]. In a similar way, Furat Rencber et al. [75] investigated the potential synergistic therapeutic effects of combined therapy of RSV and metformin on PCOS, via SIRT1 and AMPK activation. These results suggested that combined therapy of metformin and RSV may improve the weight gain, hormone profile and ovarian follicular cell architecture by inducing antioxidant and anti-inflammatory systems via SIRT1 and AMPK activation.

According to the promising results of the animal and human studies, RSV might be an effective phytochemical agent in PCOS control, especially regarding hormonal and reproductive abnormalities with influence on fertility and embryogenesis.

## 8. RSV—Future Perspectives

Although RSV has shown promising effects on a large range of pathologies, it also has pronounced limitations. The low bioavailability and its extensive metabolism are often reported as potential weak points of RSV activity.

Bioavailability studies of RSV in humans vary enormously in the choice of the administered source and in the wide range of doses applied from 5–5000 mg [76]. Additionally, there are differences in the results depending which source of RSV is tested—the alcoholic or non-alcoholic drink, some form of food or tablets. One of the first bioavailability studies of RSV showed that a single oral dose of 25 mg, which corresponds to a moderate intake of red wine, led to barely measurable concentrations of unmetabolized RSV in the circulating plasma, 10 ng/mL at 30 min to 2h after the oral used [77]. Another study used oral dose of 25 mg indicated a low plasma RSV concentration (5 nm in the first 30 min), but considerably higher concentrations of total-metabolite of RSV, around 500 ng/mL [78]. In both studies, the absorption of 25 mg oral dose of RSV was at least 70% [77,78]. Logically, studies with increasing doses of RSV were also performed, and plasma concentrations were measured. However, the studies that have examined increasing doses of 25–5000 mg of RSV have not shown the expected linearly increase in plasma RSV concentration [79]. Even after the highest dose (5000 mg), the peak plasma levels only reached about 500 ng/mL, possibly due to limited solubility. Repeated or chronic dosing might also result in saturation of metabolism, leading to a higher plasma and tissue levels of RSV [77]. Pharmacokinetics studies suggest that the intake of low-dose RSV produces a maximum peak plasma concentration within the first 30 min, while a high dose of RSV produces the maximum peak plasma concentration at 1.5–2 h [80].

Since pharmacokinetic studies indicate that RSV is rapidly metabolized [76], attention has been brought to its metabolites. Most of the oral dose appeared in urine, and liquid chromatography/mass spectrometry analysis identified three metabolic pathways—sulfate and glucuronic acid conjugation of the phenolic groups and hydrogenation of the aliphatic double bond produced by the intestinal microflora—dihydroresveratrol [78]. The reason for the limited bioavailability of RSV appears to be extremely rapid sulfate conjugation by the intestine/liver. Although the systemic bioavailability of RSV is very low, the accumulation of RSV in epithelial cells along the aerodigestive tract and potentially active RSV metabolites may still produce beneficial effects. Therefore, deconjugation enzymes such as β-glucuronidase and sulfatase, as well as specific tissue accumulation of RSV, may enhance RSV efficacy at target sites. RSV analogues, such as methylated derivatives with improved bioavailability, may be important in future research [77]. Although RSV exists naturally as both *cis*- and *trans*-isomers, most studies have used *trans*-RSV for administration due to lack of stability of the *cis* isomer, which is not commercially available for the same reason [81].

Piceid is the glycosylation product of RSV and the main storage form of stilbenes in nutrients. Piceid administration might be an alternative to pure compound intake. Burkon and Somoza suggested that piceid might be enzymatically hydrolysed in the colon or inside enterocytes, resulting in the formation of *trans*-RSV. Therefore, piceid may represent an alternative soluble form for RSV administration [82].

In this regard, the recently produced micronized RSV formulation called SRT501, shows promise. SRT501 was more bioavailable in cancer patients with no serious adverse effects, even when taken as the highest single dose of 5 g. The highest peak plasma concentration of SRT501 was 1942 ng/mL in comparison with pure RSV, which gave the highest peak plasma concentration of 538.8 ng/mL [83].

Despite these limitations, scientific and general interest in RSV has not been declining. This is supported by toxicological and side effects studies, which show very favourable results with the use of RSV. In the studies covering a period of 14 days to 3 months of RSV administration and a dose range of 15 mg to 5 g, the occurrence of toxicity of RSV has not been reported [80]. There are studies that have reported mild adverse effects such as headache, dizziness, epididymitis, nausea, diarrhea, and abdominal discomfort but only at higher doses (2.5 and 5.0 g). In obese postmenopausal women, administration of 1 g RSV for 12 weeks produced adverse effects with interindividual variations [80].

Interest in the clinical utility of RSV is constantly growing. Based on the data available on most relevant web-site for clinical trials (www.clinicaltrials.gov, accessed on 3 February 2022), 175 trials with RSV have been undertaken so far, out of which 30 are currently active. The therapeutic potential of RSV has been focusing mostly on cancer, neurological disorders, cardiovascular diseases, diabetes, non-alcoholic fatty liver disease (NAFLD), and obesity. Out of them, 12 clinical trials were investigating the effects of RSV on women’s reproductive system, and most of them were conducted on women with PCOS. Additionally, the clinical trials concerning reproductive health have been aimed to study RSV effects on endometriosis, the impact of RSV on women who have had pre-eclampsia, premature birth, hypertension and intrauterine growth retardation, as well as its influence on women fertility, or benefits of RSV in the process of IVF. Only one clinical trial focused on the impact of RSV on male fertility. Cumulatively, this can lead to a conclusion that RSV can be used in the treatment of different segments of reproductive health. However, more evidence should be provided.

## 9. Conclusions

In order to improve their health status and longevity, or to prevent perinatal morbidity and/or mortality, people often overlook simpler solutions (such as beneficial effects of proper nutrition on overall health and prevention of common diseases) by looking for some ‘synthetic, rare drugs’ conceived within laboratory settings, outside the field of nature. Nowadays, people are preoccupied by the newly developed synthetic drugs that are expanding almost daily in the public. They often forget that just by improving their diet (enriching the nutrition with natural products, such as dietary polyphenols) the burden of many chronic non-communicable diseases can probably fall below the level of significant endangerment to the public health. Besides, many children seek their exemplary behaviours and habits at home, among their parents, so majority of the habits they adopt from home can critically influence their normal growth and development, physical and psychosocial.

This paper provides comprehensive review of the effects of RSV like phytoestrogen, its effects on embryogenesis and spermatogenesis but on most common pregnancy-related complications and on umbilical blood vessels as well as women’s reproductive health overall. The protective effects of RSV are mediated through several mechanisms and include the inhibition of cell proliferation, induction of apoptosis, reduction of inflammation and damage elicited by ROS as well as smooth muscle relaxation. However, the different study designs, as well as the large variability in the dose, route and courses of RSV administration, make it difficult to reach strong conclusions about the health benefits of RSV as a phytoestrogen. Also, recent data are addressing the importance of the phase of a menstrual cycle in which RSV supplementation commences. Its restriction during the proliferative phase may promote ovarian function without further affecting embryo implantation. In contrast, RSV treatment during the initial phase may inhibit decidua transformation of the endometrium. The positive effect of RSV on the prevention of pregnancy-related complications has been observed in both pre-clinical and clinical studies. One of the examples of the beneficial effects is in the treatment of diabetic complications in pregnancy as well as relaxation of umbilical blood vessels, which reducing the risk of preeclampsia. It seems that RSV is a promising compound, can prevent or slow the progression of endometriosis, dysmenorrhea, PCOS and their symptoms. However, there are limitations regarding RSVs bioavailability and its pharmacokinetics. We hope that this overview can provide a groundwork that will encourage more detailed research at the clinical level, taking into consideration its important implications on human reproductive health.
